# Complete Genome Sequence of a Virulent Duck Enteritis Virus (DEV/India/IVRI-2016) Isolated from Southern India

**DOI:** 10.1128/mra.01244-21

**Published:** 2022-06-02

**Authors:** Satyabrata Dandapat, Suresh Bindu, Gaurav Kumar Sharma, G. Saikumar

**Affiliations:** a Indian Council of Agricultural Research-Indian Veterinary Research Institute, Izatnagar, Uttar Pradesh, India; Portland State University

## Abstract

Molecular characterization of Indian isolates of duck enteritis virus (DEV) so far has been limited to a few selected genomic regions. Here, we report the complete genome sequence of an isolate, DEV/India/IVRI-2016, from southern India that is 158,091 bp in length.

## ANNOUNCEMENT

Duck viral enteritis (DVE) or duck plague (DP) is an acute and highly contagious viral disease that is reported worldwide, with significant economic losses due to high morbidity and mortality rates (1). Duck enteritis virus (DEV), also called *Anatid herpesvirus*-*1*, is a member of the *Alphaherpesvirinae* subfamily of *Herpesviridae*, genus *Mardivirus,* an enveloped virus with a linear double-stranded DNA (dsDNA) genome of 158,091 bp (2). In India, since its first report in 1963 (3), frequent outbreaks of the disease have been reported from different parts of the country, with high mortality rates in the Indian duck populations (4–8). During a natural outbreak of DP in 2015 to 2016 in the Kuttanad region of Kerala state, India, we isolated a DEV strain (DEV/India/IVRI-2016) (GenBank accession number KX511893.1) from liver tissue samples by inoculation into embryonated duck eggs (9). Further, the pathogenicity of this isolate was checked by experimental infection in ducklings, and liver samples from the dead ducklings were used as the source of virus, from which the DNA was isolated by using the Qiagen DNeasy blood and tissue kit according to the manufacturer’s protocol.

The complete genome sequence of the isolate DEV/India/IVRI-2016 was generated using next-generation sequencing technology. Briefly, a library was prepared from the extracted DNA using a Eurofins next-generation sequencing kit, followed by paired-end Illumina TruSeq library preparation. Sequencing was performed on the NextSeq 500 system to generate 2 × 150-bp paired-end reads. Trimmomatic v0.38 software was used to remove ambiguous sequences with more than 5% unknown nucleotides N and adapter sequences. The low-quality reads of more than 10% of bases with quality threshold Phred scores of <20 was also removed to generate high-quality sequences. A total of 34,901,132 reads were generated, with 6,661,289 of them being used for alignment. The processed raw reads were aligned with the reference genome (GenBank accession number NC_013036.1) using BWA MEM v0.7.17, and the total number of aligned reads was 84,870, with a read length of 146 bp. Genome coverage was 99.99% (158,007 of 158,023 bp), which was generated relative to the reference genome (GenBank accession number NC_013036.1). All software tools were run with default parameters.

The complete genome of DEV/India/IVRI-2016 is 158,091 bp in length, with 76 coding genes and a GC content of 39.9%. BLASTn analysis revealed that the nucleotide sequence is 99% similar to the other DEV isolates available in the NCBI GenBank. This genome sequence of DEV/India/IVRI-2016 may be useful in the development and characterization of an effective vaccine candidate using the same local isolate.

### Data availability.

The genome sequence of DEV/India/IVRI-2016 has the GenBank accession number MZ824102.1, and the SRA project data are available under the SRA accession number SRR17247021.
